# Exploring the Dermatological Benefits of Coffee Extracts and Their Derivatives

**DOI:** 10.3390/antiox15020244

**Published:** 2026-02-12

**Authors:** Hsiao-Fang Liao, Po-Yuan Wu, Kuo-Ching Wen, Tsen-Jung Lin, Hung-Lung Chiang, Hsiu-Mei Chiang

**Affiliations:** 1 Ph.D. Program for Biotechnology Industry, College of Life Science, China Medical University, Taichung 406040, Taiwan; u111307801@cmu.edu.tw; 2 Department of Cosmeceutics, School of Pharmacy, China Medical University, Taichung 406040, Taiwan; kcwen0412@gmail.com; 3 Department of Dermatology, China Medical University Hospital, Taichung 404327, Taiwan; wu.poyuan@gmail.com; 4 School of Medicine, China Medical University, Taichung 404333, Taiwan; 5 School of Pharmacy, China Medical University, Taichung 406040, Taiwan; cathyletter@hotmail.com; 6 Department of Safety Health and Environmental Engineering, National Yunlin University of Science and Technology, Yunlin 604301, Taiwan

**Keywords:** coffee, dermatology, skin aging, anti-inflammatory, wound healing, skin cancer

## Abstract

Coffee-derived materials from diverse botanical sources (beans, leaves, fruit and spent grounds) contain bioactive polyphenolic compounds, alkaloids, and diterpenes with potential dermatological applications. This review critically evaluates evidence quality across study designs. *In vitro* studies demonstrate antioxidant, anti-aging, anti-inflammatory, photoprotective, wound-healing, and antimicrobial activities. Animal models show photoprotection and wound-healing effects. These studies highlight the multifunctional dermatological value of coffee-derived materials as ingredients for cosmetic and therapeutic formulations aimed at combating skin aging, inflammation, and barrier dysfunction. Limited human trials (typically small sample sizes and short duration) report modest improvements in skin hydration, elasticity, barrier function, and reductions in erythema, transepidermal water loss, and ultraviolet-induced damage, though methodological limitations constrain interpretation. Observational epidemiological studies report inverse associations between coffee consumption and melanoma/basal cell carcinoma risk, but residual confounding by sun exposure, lifestyle factors, and genetic susceptibility precludes causal inference. Critical translational barriers include insufficient pharmacokinetic characterization, inadequate extract standardization across sources and processing methods, formulation challenges, bioavailability uncertainties, and limited independent validation. While preclinical evidence supports diverse biological activities and suggests multifunctional potential for cosmetic and therapeutic applications, current evidence remains insufficient to recommend coffee-derived products as a primary evidence-based dermatological intervention. Overall, large-scale, independent clinical trials with adequate duration and clinically meaningful endpoints are essential for translating laboratory findings into validated clinical applications.

## 1. Introduction

Coffee is one of the most widely consumed beverages worldwide and the coffee industry generates substantial biomass across multiple processing stages. While coffee beans remain the primary commercial product, the coffee plant and its processing by-products—including leaves, berry pulp, silverskin, and spent coffee grounds—represent largely underutilized resources with notable phytochemical richness and therapeutic effects [1,2]. These coffee-derived materials exhibit diverse phytochemical profiles depending on their botanical origin, processing methods, and extraction conditions, with applications spanning from topical formulations to oral supplements. Traditional coffee-growing regions, including Ethiopia, Indonesia, and South America, have long incorporated coffee leaves into medicinal practices, preparing tea-like infusions valued for their phytonutrient content and ethnomedicinal properties [3].

Coffee-derived materials present distinct and complementary phytochemical profiles depending on their botanical source and processing procedures. Phytochemical investigations indicate that the coffee plant contains a range of bioactive compounds relevant to dermatological research. Across different plant parts, coffee-derived materials have been reported to contain polyphenols, alkaloids, and flavonoids associated with antioxidative and anti-inflammatory activities. Coffee beans contain high concentrations of chlorogenic acids, 5-caffeoylquinic acid, along with trigonelline and diterpenes. Coffee leaves have been described as containing chlorogenic acids, mangiferin, and various flavonoids [4,5,6], with some studies reporting higher total phenolic content compared with green tea or coffee beans [7,8]. Coffee fruit (pulp and skin) contains anthocyanins, proanthocyanidins and phenolic acids. In addition, spent coffee grounds retain appreciable levels of melanoidins, fiber-bound polyphenols, and lipophilic compounds. Each part of coffee plant offers various dermatological application potential warranting comprehensive evaluation.

Skin health and aging are significantly influenced by oxidative stress, chronic inflammation, and environmental exposures, particularly ultraviolet (UV) radiation, which collectively contribute to photoaging inflammatory skin disorders, and carcinogenesis [4,5]. Coffee-derived materials have demonstrated dermatological activities by modulating key molecular pathways involved in oxidative damage, inflammatory signaling, extracellular matrix (ECM) degradation, and cellular repair processes [6,7,8]. In addition to coffee leaf, coffee berry extract demonstrates anti-aging effects through superoxide dismutase modulation and collagenase inhibition in human dermal fibroblasts [9,10]. Spent coffee ground extracts reduced UVB-induced photoaging via downregulation of matrix metalloproteinase (MMP) [11]. Consumption of coffee polyphenol from green coffee may improve skin barrier function, reducing transepidermal water loss (TEWL) while enhancing stratum corneum hydration [12]. These findings suggest coffee-derived materials and coffee by-products may be used as cosmeceutical ingredients. This review examines coffee-derived materials in dermatology, analyzing evidence quality and bioavailability considerations, and discusses translational challenges that must be addressed before clinical applications can be recommended.

This review aims to evaluate current studies regarding the dermatological applications of coffee-derived materials across diverse source materials (beans, leaves, fruits and spent grounds). We assess evidence quality across study designs (*in vitro* studies, animal models, and human clinical trials), examine dose–response relationships and bioavailability considerations, evaluate methodological limitations and identify critical research gaps. We examine the classification and quantification of major bioactive compounds in coffee-derived materials and assess their therapeutic effects across multiple domains: (1) anti-aging and photoprotective mechanisms; (2) anti-inflammatory effects and potentially use in inflammatory dermatoses; (3) wound-healing and tissue-regeneration activity; (4) epidemiological evidence supporting coffee consumption is associated with low risk of skin cancer; (5) additional effects including skin-lightening, antimicrobial, and anti-hair-loss properties; and (6) clinical studies evaluating the impact of coffee extracts on human skin. We also identify critical research gaps and provide recommendations for future investigation. By integrating evidence across study designs and addressing limitations and translational challenges, this review seeks to offer a balanced appraisal of current knowledge and a realistic assessment of clinical application potential.

## 2. Major Active Compounds in Coffee-Derived Extracts

Coffee-derived extracts contain a diverse array of bioactive compounds that can be broadly classified into four major categories based on quantitative distribution. These constituents play key roles in the antioxidative and anti-aging activities associated with coffee-derived extracts.

### 2.1. Polyphenolic Compounds

#### 2.1.1. Xanthones

Mangiferin is the most distinctive xanthone found in coffee leaves and is particularly abundant in *Coffea arabica*, occurring at concentrations ranging from 0.6 to 5.0 mg/g in dry weight. Among 23 *Coffea* species analyzed, seven accumulate mangiferin, with *Coffea pseudozanguebariae* exhibiting the highest levels. Isomangiferin is also present but in substantially lower quantities, typically representing 5–10 mg/g of mangiferin content in extracts [13,14,15].

#### 2.1.2. Chlorogenic Acids

Chlorogenic acids constitute the largest class of phenolic compounds in coffee leaves and coffee bean extracts [16]. The predominant form, 5-caffeoylquinic acid, is present at 15.2–42.3 mg/g in dry weight, followed by 3-caffeoylquinic acid (2.5–8.7 mg/g) and 4-caffeoylquinic acid (1.8–6.2 mg/g). Feruloylquinic acids occur at 0.5–3.1 mg/g, while dicaffeoylquinic acids range from 1.2 to 7.3 mg/g depending on isomeric form and species [14,17,18].

#### 2.1.3. Flavonoids

Coffee leaves contain a range of flavonoids, including rutin and its isomers, quercetin derivatives, kaempferol glycosides, catechin, and epicatechin [19].

#### 2.1.4. Procyanidins

Procyanidin B and procyanidin C have been identified in coffee left extracts and contribute to the antioxidant capacity of the plant [19].

### 2.2. Alkaloids

#### 2.2.1. Purine Alkaloids

Caffeine concentrations in coffee leaves are notably lower than in coffee beans, ranging from 0.5 to 1.8 mg/g in *Coffea arabica* leaves and 0.8 to 2.3 mg/g in *Coffea canephora* leaves. Young leaves contain two- to three-fold higher caffeine concentrations than mature leaves [15,20].

#### 2.2.2. Pyridine Alkaloids

Trigonelline is present at substantial concentrations, ranging from 60 to 180 μmol/g (equal to 8.23–24.69 mg/g) dry weight. The compound accumulates most heavily in young stems and buds (up to 180 μmol/g), with moderate levels observed in leaves (60–80 μmol/g) [20,21].

### 2.3. Terpenoids

Coffee leaves contain diterpenes such as cafestol, 16-O-methylcafestol, and kahweol, along with essential oils composed of monoterpenes, sesquiterpenes, and oxygenated derivatives [22].

## 3. Methodology for Literature Selection

The peer-reviewed literature on coffee-derived compounds in dermatological applications were searched for on the PubMed and Web of Science databases. The time frame is focused between May 2010 and 2024. The following keywords and their combinations were used: “coffea,” “coffee,” “skin,” “epiderm*,” “derm*,” and “cutaneous.” These terms were selected to ensure the inclusion of all relevant dermatological studies involving coffee plant extracts and related components. After removing duplicate entries and non-English publications, articles were screened for relevance based on their focus on the dermatological effects of coffee-derived materials. Inclusion criteria emphasized studies addressing anti-aging, anti-inflammatory, wound-healing, skin-cancer-preventive effects, and improvements in other dermatological parameters. Findings are presented according to the specific dermatological benefits associated with coffee plant extracts.

## 4. Antioxidative and Anti-Aging Effects

Skin aging is broadly classified into intrinsic and extrinsic processes [23,24], both of which lead to visible alterations in cutaneous appearance and structure. Intrinsic aging represents the natural degenerative process characterized by dryness, reduced elasticity, and fine wrinkles, primarily attributed to decreased collagen production and flattening of the dermal–epidermal junction [25]. In contrast, extrinsic aging results from environmental stressors, particularly UV exposure, and manifests as coarse wrinkles, rough texture, and pigmentation irregularities, predominantly on sun-exposed areas such as the face, neck, and hands [26]. A key mechanism underlying skin aging involves reduced collagen biosynthesis and increased activity of matrix metalloproteinase (MMPs), which degrade collagen fibers. Reactive oxygen species (ROS) generated by both intrinsic and extrinsic factors activate transcription factors such as activator protein 1 (AP-1), leading to MMP upregulation and suppression of transforming growth factor-β (TGF-β) signaling. This results in impaired ECM integrity and contributes to a feedback loop wherein aging fibroblasts generate more ROS, further accelerating dermal aging [24,27]. Coffee leaves were investigated and showed superior antioxidant capacity compared to roasted beans, the young leaves exhibiting the highest total phenolic content and antioxidant capacity [28]. Coffee leaves exhibited significantly higher antioxidant activities than green beans in both 2,2′-diphenyl-1-picrylhydrazyl (DPPH) and 2,2′-azino-bis-3-ethylbenzothiazoline-6-sulfonic acid (ABTS) assays, with anti-inflammatory effects through ROS, nitric oxide (NO), and nuclear Factor kappa-light-chain-enhancer of activated B cell (NF-κB) inhibition [29]. The *Coffea arabica* leaf extracts reduced the O_2_^•-^, ROO^•^, H_2_O_2_ and HOCl reactive oxygen species. In addition to ROS-mediated pathways, UV irradiation directly induces DNA damage such as cyclobutane pyrimidine dimers (CPDs) and pyrimidine–pyrimidone photoproducts, which activate cellular stress responses and further drive photoaging phenotypes [30,31].

The anti-aging effects of coffee-derived materials in cellular and animal models are summarized in Table 1. *Coffea arabica* leaf extract has demonstrated significant protective effects against UVB-induced damage in Hs68 fibroblasts and UVB-irradiated mouse models [6,17]. Treatment inhibited MMP expression, restored type I procollagen levels, and decreased MAPK phosphorylation, thereby preserving collagen structure and reducing ECM degradation [17]. Additionally, the extract inhibited collagenase-1 and elastase, two enzymes associated with wrinkle formation and loss of elasticity [17]. In UVB-irradiated BALB/c mice, *Coffea arabica* leaf extract also reduced intracellular ROS, enhanced catalase activity, improved dermal collagen density in the dermis, and inhibited MMP-1 levels, further supporting its photo-protective properties [6].

Extracts obtained from spent coffee grounds have similarly demonstrated anti-aging activity [11,32,33,34]. Oral administration of spent coffee ground extract in UVB-irradiated mice reduced wrinkle formation, epidermal thickening, and TEWL [32], while decreasing MMP-2 and MMP-9 expression and restoring type I collagen, indicating an improvement in skin structure and resilience [32]. Topical application of oil and ethanol extracts yielded comparable improvements in wrinkles, epidermal thickness, erythema, and hydration, accompanied by downregulation of MMP-2, -9, and -13 expression [11]. *In vitro* studies have also shown that spent coffee ground extract reduced intracellular ROS and inhibited elastase activity in keratinocytes, indicating its protective role against oxidative stress and enzymatic degradation of skin components [33]. Moreover, spent coffee ground oil increased collagen production and inhibited MMP-2 activity in normal human skin fibroblasts, further supporting its relevance in improving skin structure and integrity [34].

Green coffee-derived extracts have also demonstrated antioxidant and anti-aging activity in cellular models [35,36,37]. Green coffee kombucha ferments applied to HaCaT keratinocytes and BJ fibroblasts effectively suppressed ROS generation and inhibited collagenase and elastase activity, indicating potential effects against oxidative stress and enzymatic degradation of skin components [35]. Similarly, green coffee bean extract reduced elastase activity in mouse fibroblasts, providing further protection against collagen degradation [36]. Coffee cherry pulp and berry extracts demonstrated significant anti-aging and antioxidant effects in cellular models [10,38]. Coffee cherry pulp extract effectively inhibited the activities of collagenase, elastase, and hyaluronidase in keratinocytes and reduced intracellular ROS [38]. Nanoliposomes-encapsulated coffee berry extract further enhanced superoxide dismutase (SOD) activity, reduced NO levels, and inhibited collagenase in human dermal fibroblasts, further enhancing the skin’s antioxidant defense and mitigating aging markers [10].

**Figure 1:**
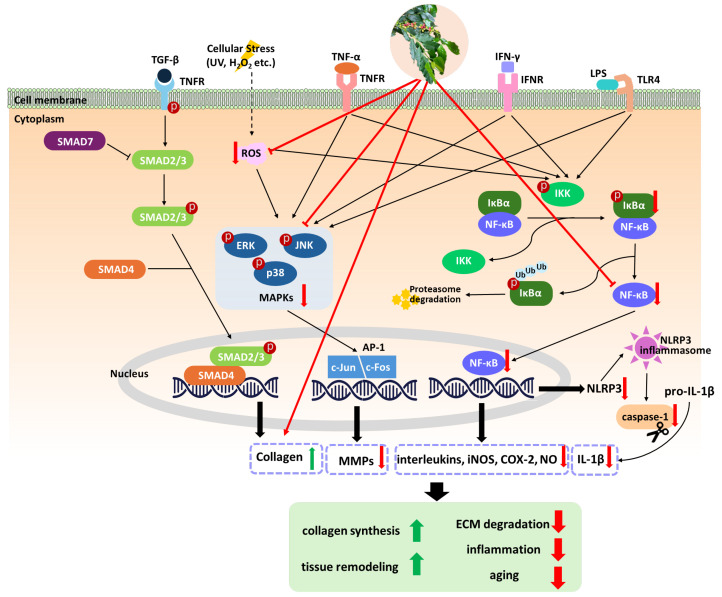
Molecular mechanisms of coffee extracts in mitigating oxidative stress and inflammation. UV radiation, pollutants, and aging induce reactive oxygen species (ROS), triggering mitogen-activated protein kinase (MAPK, including ERK/JNK/p38) signaling and activator protein-1 (AP-1)-mediated upregulation of matrix metalloproteinase (MMPs), leading to extracellular matrix (ECM) degradation. Coffee polyphenols neutralize ROS and boost antioxidant enzymes (superoxide dismutase (SOD), glutathione peroxidase (GPx), catalase), inhibiting the MAPK/AP-1 axis and preserving collagen via transforming growth factor beta (TGF-β)/SMAD signaling. Concurrently, coffee compounds suppress nuclear factor kappa-light-chain-enhancer of activated B cell (NF-κB) nuclear translocation by preventing inhibitor of kappa B (IκB) degradation, inhibit NOD-like receptor protein 3 (NLRP3) inflammasome/caspase-1 maturation of IL-1β, and modulate upstream receptors such as toll-like receptor 4 (TLR4) and tumor necrosis factor/interferon receptor (TNFR/IFNR), collectively reducing pro-inflammatory mediators (tumor necrosis factor alpha (TNF-α), interleukins (ILs), inducible nitric oxide synthase (iNOS) and cyclooxygenase-2 (COX-2). 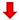, decrease; 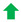, increase.

Coffee silverskin extract has also shown anti-aging properties in both cellular and organismal models [39]. In HaCaT keratinocytes, the extracts maintain cell viability and protect against oxidative stress [39]. Furthermore, in *Caenorhabditis elegans*, the extract extended lifespan, suggesting broader modulatory effects on aging pathways. Collectively, these findings highlight the diverse effects of coffee silverskin extract as a natural agent for enhancing cellular health and longevity [39]. Each study showcases the beneficial effects of different coffee-derived materials on reducing skin aging, whether through antioxidative mechanisms, inhibiting collagen-degrading enzymes, or by improving skin structure through collagen content restoration. The proposed mechanisms by which coffee extracts mitigate skin aging are illustrated in Figure 1.

Overall, evidence supporting the antioxidative and anti-aging effects of coffee-derived materials is largely derived from *in vitro* and animal studies, with relatively limited and heterogeneous human data, indicating that while mechanistic support is substantial, clinical translation remains preliminary.

**Table 1:** Anti-aging effects of coffee-derived materials in cellular and animal models.

Material	Experimental Model	Experimental Design		Main Findings	Reference
		Inducer	Route of Administration		
*Coffea arabica* leaf extract	Hs68 cells	UVB	-	Inhibited MMP-1, -3, -9 expression, restored type I procollagen, and reduced phosphorylation of MAP kinases (ERK, JNK and p38) Inhibited collagenase-1, elastase activity and reduced gelatin digestion	[17]
Spent coffee ground extract	SKH-1 hairless mice	UVB	Oral administration	Reduction in wrinkles Decrease in epidermal thickness, TEWL and erythema Suppression of MMP-2 and MMP-9 expression Restored type 1 collagen	[32]
Spent coffee ground extract oil and ethanol extract	SKH-1 hairless mice	UVB	Topical application	Reduction in wrinkles, epidermal thickness and erythema formation Increased water-holding capacity Decreased the mRNA levels of MMP-2, -9, and -13 Restored type 1 collagen	[11]
Coffee silverskin extract	HaCaT cells	*t*-BOOH	-	Maintained cell viability and reduced intracellular ROS levels	[39]
	*C. elegans*	UVC	-	Increased the lifespan of *Caenorhabditis elegans*	
*Coffea arabica* leaf extract	Hs68 cells	UVB	-	Reduced intracellular ROS levels and enhanced CAT activity	[6]
	BALB/c hairless mice	UVB	Topical application	Restored collagen content in the dermis Inhibited MMP-1 levels	
Spent coffee ground extract	HaCaT cells	H_2_O_2_	-	Reduced intracellular ROS levels Inhibited elastase	[33]
Green coffee kombucha ferment	HaCaT cells/ BJ cells	H_2_O_2_	-	Reduced ROS generation Inhibited collagenase and elastase activities	[35]
Green coffee bean (*Coffea* *arabica* L.) extract	L929 cells	UV	-	Inhibited elastase activity	[36]
Spent coffee ground oil	Normal human skin fibroblasts	-	-	Enhanced collagen production Inhibited MMP-2 activity	[34]
Coffee cherry pulp extract	HaCaT cells	polycyclic aromatic hydrocarbon (PAH)	-	Inhibited collagenase, elastase, and hyaluronidase enzymes Reduced intracellular ROS levels	[38]
Coffee berry extract (nanoliposome)	Human Dermal Fibroblasts	Nitric oxide	-	Increased SOD activity Inhibited nitric oxide production and collagenase	[10]

Abbreviation: catalase (CAT); reactive oxygen species (ROS); matrix metalloproteinase (MMP); mitogen-activated protein kinase (MAP kinase); superoxide dismutas (SOD); transepidermal water loss (TEWL).

## 5. Anti-Inflammatory Effects

Cutaneous inflammation is a complex immune-mediated defense mechanism activated in response to external stimuli such as UV radiation, pathogens, and environmental stressors. While these responses are essential for host protection and maintaining skin homeostasis, dysregulation inflammation can lead to chronic inflammatory skin disorders, including atopic dermatitis and psoriasis [40]. These conditions are characterized by persistent inflammation, tissue damage, and impairment of the skin barrier. A central pathway involved in skin inflammation is the IκB kinase/NF-κB signaling pathway, which plays a dual role in regulating immune responses. Increased activation of NF-κB is frequently observed in immune and non-immune cells within inflamed skin [41,42], resulting in excessive production of pro-inflammatory cytokines and chemokines that perpetuate the chronic inflammation. In atopic dermatitis, for instance, elevated NF-κB activity promotes the release of inflammatory mediators such as IL-4, IL-13, and TNF-α, amplifying the inflammatory cascade and exacerbating disease severity [43].

Several studies have demonstrated anti-inflammatory properties of coffee-derived materials (Table 2). *Coffea arabica* leaf extract has demonstrated notable anti-inflammatory effects in various experimental models, including cellular and animal studies [6]. In Hs68 fibroblasts and UVB-irradiated BALB/c hairless mice, the extract effectively inhibited cyclooxygenase-2 (COX-2) expression and suppressed the activation of the IκB/NF-κB signaling pathway, while reducing erythema, TEWL, and epidermal hyperplasia. It also significantly decreased expression of pro-inflammatory mediators such as inducible nitric oxide synthase (iNOS), IL-6, and NF-κB, indicating robust protection against UV-induced cutaneous inflammation and damage [6]. In Swiss albino mice, *Coffea arabica* leaf extract significantly reduced ear edema induced by croton oil, phenol, and histamine by inhibiting myeloperoxidase (MPO) and N-acetyl-β-D-glucosaminidase (NAG) activity [44], suggesting broad-spectrum anti-inflammatory activity across different inflammatory stimuli. Furthermore, in HaCaT keratinocytes and in 2,4-dinitrochlorobenzene (DNCB)-induced atopic dermatitis mouse models, the extract reduced ROS production, inhibited nuclear of NF-κB translocation, and decreased secretion of IL-1β and IL-6 [45]. It also downregulated NOD-like receptor pyrin domain-containing protein 3 (NLRP3) and caspase-1 expression, restored barrier-related proteins (filaggrin and claudin-1), reduced epidermal thickness and erythema, and lowered TEWL, TNF-α, and thymic stromal lymphopoietin (TSLP) [45]. Collectively, these findings support the therapeutic relevance of *Coffea arabica* leaf extract in managing inflammatory dermatoses, including atopic dermatitis, by modulating key inflammatory pathways and enhancing skin barrier function.

Coffee cascara kombucha demonstrated anti-inflammatory activity by reducing nitric oxide production in RAW 264.7 macrophages, suggesting its ability to modulate inflammatory responses at the cellular level [46]. Similarly, *Coffea canephora* leaf-derived stem cell extracts reduced superoxide anion, nitric oxide, TNF-α, and IL-6 production in RAW 267.2 macrophages and L929 fibroblasts, while simultaneously promoting cell proliferation and migration, suggesting concurrent anti-inflammatory and wound-healing benefits [8]. Additionally, green coffee bean extracts reduced carrageenan-induced edema in Wistar rats and decreased inflammatory markers including Langerin, S100, and α-SMA [47], further underscoring its effect to mitigate cutaneous inflammation and maintain skin homeostasis. These studies indicate that coffee-derived extracts can modulate key inflammatory pathways involved in cutaneous inflammation and barrier dysfunction. In addition, NF-κB pathway inhibition is consistently observed in keratinocytes and macrophage models across studies, with corresponding reductions in pro-inflammatory cytokines including IL-6, IL-8 and TNF-α. However, the current evidence is primarily derived from *in vitro* and animal models, and clinical data remain limited, underscoring that translational relevance to human inflammatory dermatoses requires cautious interpretation. The proposed mechanisms through which coffee extracts mitigate inflammatory pathways are illustrated in Figure 1.

**Table 2:** Anti-inflammatory effects of coffee-derived materials in cellular and animal models.

Materials	Experimental Model	Experimental Design		Main Findings	Reference
		Inducer	Route of Administration		
*Coffea arabica* leaf extract	Hs68 cells	UVB	-	Inhibited COX-2 expression and IκB/NF-κB signaling cascade	[6]
	BALB/c hairless mice	UVB	Topical application	Reduced skin erythema, TEWL, and epidermal hyperplasia Inhibited iNOS, IL-6, and NF-κB levels	
*Coffea arabica* leaf extract	Swiss albino mice	Croton oil Phenol Histamine	Topical application	Inhibited MPO and NAG Reduced mouse ear edema	[44]
Green coffee bean (*Coffea robusta*) extract	Wistar rats	Carrageenan	Topical application	Inhibition of edema formation decreased expression of Langerin, S100 and α-SMA	[47]
*Coffea arabica* leaf extract	HaCaT cells	TNF-α/ IFN-γ	-	Reduced ROS generation Inhibited the translocation of NF-κB into the nucleus Reduced IL-1β and IL-6 secretion Decreased the expression of NLRP3 and caspase-1 Improved filaggrin and claudin-1	[45]
	BALB/c mice	DNCB	Topical application	Reduced epidermal thickness, redness and TEWL Decreased the levels of TNF-α and TSLP	
Coffee cascara kombucha	RAW 264.7 macrophages	LPS	-	Reduced nitric oxide formation	[8]
Stem cell extracts from *Coffea canephora* leaves	RAW 267.2 macrophages L929 cells	LPS	-	Suppressed superoxide anion, nitric oxide, TNF-α and IL-6 Promoted the proliferation and migration	[46]

Abbreviation: cyclooxygenase-2 (COX-2); inhibitor of nuclear factor kappa B (IκB); lipopolysaccharide (LPS); myeloperoxidase (MPO); N-acetyl-β-D-glucosaminidase (NAG); nuclear factor kappa-light-chain-enhancer of activated B cells (NF-κB); tumor necrosis factor-α (TNF-α).

## 6. Wound-Healing Effects

Wound healing is a complex, dynamic, and highly regulated biological process essential for restoring tissue integrity and function following injury. It progresses through four overlapping phases: (i) coagulation and hemostasis, (ii) inflammation, (iii) proliferation, and (iv) remodeling [48].

Several studies, summarized in Table 3, have demonstrated the beneficial effects of coffee-derived materials on wound healing and tissue repair. Topical application of coffee oils in Sprague–Dawley (SD) rats significantly accelerated wound closure and increased collagen density, suggesting enhanced tissue repair and improved structural integrity of the regenerated skin [49]. Similarly, green coffee oil promoted proliferation and migration, essential processes for wound closure and re-epithelialization, in human epidermal keratinocytes [50]. Moreover, topical application of green coffee bean extract to SD rats accelerated wound healing and increased the expression of key regenerative markers such as fibronectin and fibroblast growth factor (FGF), both essential for tissue repair [51]. Topical application of *Coffea arabica* bean residual press cake in Swiss albino mice also resulted in reduction of wound area, further supporting the association of coffee-derived materials with wound-healing effects [52].

**Table 3:** Wound-healing effects of coffee-derived materials in cellular and animal models.

Materials	Experimental Model	Route of Administration	Main Findings	Reference
Coffee (*Coffea arabica* L.) bean residual press cake	Swiss albino mice	Topical application	Reduced the wound area	[52]
Coffee oils	SD rats	Topical application	Accelerated wound healing Increased collagen density	[49]
Green coffee bean extract	SD rats	Topical application	Accelerated wound healing Increased fibronectin and FGF expression	[51]
Green Coffee Oil	HaCaT cells	-	Enhanced the proliferative and migratory capacity	[50]
Liposomal formulation of *Coffea canephora* stem cell extract	Wistar rats	Topical application	Reduced wound area and inflammatory infiltrate Decreased TNF-α and IL-6 and increased IL-10 and TGF-β levels Increased collagen production and deposition	[53]
Chitosan film containing *Coffea arabica* leaf extract	L929 cells	-	Promoted cell migration	[54]

Two studies have evaluated formulated coffee-derived products. A liposomal formulation containing *Coffea canephora* stem cell extract applied to Wistar rats reduced wound size and inflammatory cell infiltration, while modulating cytokine levels by reducing pro-inflammatory TNF-α and IL-6 and increasing anti-inflammatory IL-10 and TGF-β [53]. This formulation also promoted collagen production and deposition, indicating comprehensive anti-inflammatory and pro-regenerative activity. Similarly, a chitosan film loaded with *Coffea arabica* leaf extract enhanced fibroblast migration in mice, a critical step in wound closure [54]. These findings collectively demonstrate the therapeutic relevance of coffee-derived extracts in enhancing wound healing through anti-inflammatory activity, enhanced cell proliferation, and improved tissue regenerative mechanisms. The antioxidant and anti-inflammatory properties that may promote skin regeneration to promote wound healing, and the applications of coffee leaf extracts in wound-healing scaffolds and bioactive dressings need further study.

While current research on coffee extracts in dermatology provides a strong foundation, there are opportunities to enhance its clinical relevance. Moving beyond immortalized cell lines and rodent models toward 3D human skin equivalents could better reflect physiological realities. Additionally, standardizing extraction methods and concentrations will be essential for improving the consistency and reproducibility of findings across the field. Overall, evidence supporting the wound-healing effects of coffee-derived extracts is predominantly based on animal models, while well-controlled human studies remain scarce, limiting direct clinical translation at present.

## 7. Epidemiological Associations Between Coffee Consumption and Skin Cancer Risk

Epidemiological studies have explored associations between coffee consumption and skin cancer risk, particularly melanoma and basal cell carcinoma, as summarized in Table 4. Caffeine is hypothesized to be a key bioactive compound contributing to this preventive effect. While several observational studies report inverse associations, it is important to recognize that such evidence cannot establish causation and should be interpreted with appropriate methodological considerations regarding potential confounding factors, unmeasured variables, and methodological limitations.

The European Prospective Investigation into Cancer and Nutrition (EPIC) study [55] followed 476,160 individuals across 10 European countries for a median of 14.9 years and recorded 2712 melanoma cases. Caffeinated coffee consumption was associated with lower melanoma risk in men (highest intake vs. none: hazards ratio [HR] = 0.31, 95% confidence interval [CI]: 0.14–0.69); however, this association was not observed in women (HR = 0.96, 95% CI: 0.62–1.47). This effect was primarily driven by caffeinated coffee, as decaffeinated coffee showed no significant association with melanoma risk [55]. The association appeared stronger for melanomas on the head/neck (HR = 0.56, 95% CI: 0.34–0.91) and trunk (HR = 0.85, 95% CI: 0.76–0.96). The sex-specific differences may reflect variations in sun exposure patterns, hormonal factors, or other lifestyle variables that warrant further investigation.

Similar findings were reported in the NIH-AARP Diet and Health Study [56] and an Italian case–control study [57]. The U.S. cohort analysis included 447,357 non-Hispanic White participants and documented 2904 incident melanoma cases over 4.3 million person-years of follow-up. The analysis showed lower melanoma risk among consumers compared with non-consumers, that is, participants who consumed ≥4 cups of coffee daily (HR = 0.80, 95% CI: 0.68–0.93, p-trend = 0.01). This association was significant only for caffeinated coffee (≥4 cups/day: HR = 0.75, 95% CI: 0.64–0.89) and not for decaffeinated coffee [56]. Higher caffeine intake was also associated with modestly reduced melanoma risk (HR = 0.90, 95% CI: 0.80–0.99) [56]. A hospital-based case–control study in Italy involving 304 melanoma cases and 305 controls reported that frequent coffee intake (>1 time/day) was associated with lower melanoma risk (odds ratio [OR]: 0.46, 95% CI: 0.31–0.68), after adjustment for established melanoma risk factors including sex, age, education, hair color, skin phototype, common nevi, and childhood sunburn history [57]. Notably, the association appeared stronger among individuals carrying both GSTM1 and GSTT1 null polymorphisms, suggesting potential genetic modulation of the coffee-melanoma relationship, possibly through glutathione S-transferase-mediated metabolism of coffee phytochemicals [57].

In a pooled analysis of three large U.S. cohorts (NHS II, NHS, and HPFS), including 203,310 participants with 2254 incident melanoma cases, high caffeine intake (≥393 mg/day) was associated with reduced melanoma risk (HR = 0.78, 95% CI: 0.64–0.96). The association was stronger in women (≥393 mg/day: HR = 0.70, 95% CI: 0.58–0.85) compared with men (HR = 0.94, 95% CI: 0.75–1.2) [58]. Interestingly, the association was more pronounced for melanomas on chronically sun-exposed sites (head, neck, extremities; HR = 0.71, 95% CI: 0.59–0.86) relative to trunk melanomas (HR = 0.90, 95% CI: 0.70–1.2), which may suggest interaction with UV-related pathways, though this requires further mechanistic investigation [58].

Beyond melanoma, basal cell carcinoma (BCC) represents another highly prevalent skin cancer, and has also been examined in relation to coffee consumption. A study involving 767 participants under 40 years reported that combined consumption of caffeinated coffee and hot tea was associated with reduced early-onset BCC risk (OR: 0.60, 95% CI: 0.38–0.96). Individuals in the highest category of caffeine consumption showed lower BCC risk (OR: 0.57, 95% CI: 0.34–0.95) [59], providing additional observational data suggesting potential associations between caffeine-containing beverages and skin cancer risk. These studies suggest that coffee consumption was associated with lower risk of the incidence of skin cancer.

These epidemiological findings seem to demonstrate positive results of coffee consumption with cancer prevention; however, they must be interpreted with caution due to study design or data collection. Observational studies are inherently susceptible to confounding by factors that may differ systematically between coffee consumers and non-consumers. These include sun-related behaviors (outdoor activities, sun protection practices and tanning habits), skin characteristics (phototype and freckling), and broader lifestyle factors (diet quality, smoking history, physical activity levels and healthcare access). While most analyses adjusted for known variables, residual and unmeasured confounding remains a plausible explanation for the observed effects. This is further underscored by the sex-specific inconsistency (a protective association observed in men but not women), which suggests that biological sex may interact with complex hormonal, metabolic, or behavioral factors. The possibility of reverse causation cannot be dismissed, as individuals with high-risk profiles may have proactively altered their dietary habits prior to enrollment. These findings carry significant public health relevance, particularly given the rising global incidence of melanoma and the widespread consumption of coffee. From a mechanistic perspective, the biological plausibility of coffee’s anti-carcinogenic effects requires further validation. While some *in vitro* and animal studies suggest potential photoprotective and antioxidant mechanisms, whether the concentrations achieved through typical dietary consumption are sufficient to exert clinically meaningful effects in human skin remains unclear. The relationship between coffee consumption patterns, bioactive constituent bioavailability, and tissue-level exposure requires better characterization. Overall, the epidemiological evidence linking coffee consumption to reduced skin cancer risk is derived exclusively from observational studies and should therefore be considered hypothesis-generating rather than confirmatory, with causal inference limited by residual confounding, exposure misclassification, and lack of mechanistic validation in humans. Further research is warranted to confirm these relationships and to identify the specific bioactive constituents responsible for the protective effects.

**Table 4:** Epidemiologic studies on coffee consumption on skin cancer risk.

Population (Case/Control)	Intervention	Comparison	Outcome	Reference
Cutaneous melanoma (188/152)	High-frequency coffee consumption (more than once daily)	Low-frequency coffee consumption and different GSTM1 and GSTT1 genotypes	The primary outcome measured is the risk of developing cutaneous melanoma. Reduced risk of cutaneous melanoma, especially in those with null polymorphisms for GSTM1 and GSTT1.	[57]
Basal cell carcinoma (377/390)	Regular consumption of caffeinated coffee and hot tea	No consumption or lower frequency of caffeinated coffee and hot tea	The primary outcome measured is the risk of early-onset basal cell carcinoma Reduced risk of early-onset basal cell carcinoma	[59]
Participants from three large cohort studies	Higher total caffeine intake and caffeinated coffee consumption	Lower caffeine intake and non-consumption or lower consumption of caffeinated coffee	Risk of cutaneous malignant melanoma Decreased risk of cutaneous malignant melanoma, particularly in women and on body sites with higher continuous sun exposure	[56]
447,357 participants	Consuming four or more cups of coffee per day	Consuming fewer than four cups of coffee per day or no coffee at all	The primary outcome measured was the incidence of malignant melanoma Decreased risk of malignant melanoma by 20%	[58]
Over 500,000 individuals	Consumption of caffeinated coffee, decaffeinated coffee, and tea	Non-consumption or lower consumption of caffeinated coffee, decaffeinated coffee, and tea	The primary outcome measured was the incidence of melanoma Reduced risk of melanoma associated with caffeinated coffee consumption among men, with no significant associations for women, decaffeinated coffee, or tea	[55]

## 8. Additional Skin-Related Effects of Coffee Extracts

Coffee-derived materials exhibit several additional dermatological benefits, as shown in Table 5. Spent coffee extracts have demonstrated skin-whitening activity in B16F10 melanoma cells by inhibiting melanin synthesis and inhibiting tyrosinase and tyrosinase-related protein-2 (TRP-2) activities, supporting their effect as natural depigmenting agents [34,60]. Coffee pulp extract enhanced dermal papilla cell proliferation and migration while downregulating *SRD5A1–3* gene expression, indicating relevance in preventing androgen-related hair loss [61].

Arabica coffee extract displayed broad-spectrum antibacterial activity against Gram-positive and Gram-negative bacteria, with lower minimum inhibitory concentration (MIC) values for *Staphylococcus aureus* and *Staphylococcus epidermidis*, independent of caffeine content [62]. Spent coffee ground extracts also exhibited potent antifungal effects against *Candida krusei*, *Candida parapsilosis*, *Trichophyton mentagrophytes*, and *Trichophyton rubrum* by reducing ergosterol, chitin, and β-(1,3)-glucan synthesis, compromising fungal cell wall integrity [63]. However, such broad-spectrum antimicrobial activity may not always be advantageous, as excessive suppression of commensal skin bacteria could disrupt microbial homeostasis and potentially create ecological niches favorable to opportunistic pathogens [64]. Therefore, future studies should assess the selectivity of coffee-derived extracts toward pathogenic organisms versus beneficial microbiota.

Additionally, green *Coffea arabica* seed oil improved skin photoprotection in UV-irradiated mice by reducing TEWL, sunburn cell formation, and erythema [65]. These findings indicate that coffee-derived materials are associated with a range of additional skin-related activities across experimental models. However, the available evidence for these effects is largely limited to *in vitro* and animal studies, with minimal clinical validation, and their translational relevance remains constrained by uncertainties regarding dose relevance, selectivity, and long-term safety.

**Table 5:** Other effects of coffee-derived materials on skin.

Effects	Materials	Experimental Model	Experimental Design	Main Findings	Reference
Whitening	Spent Coffee extract (100, 300 mg/mL)	B16F10 melanoma cells	Inducer: α-MSH	Inhibited melanin synthesis and tyrosinase activity	[60]
Whitening	Spent coffee ground oil	B16F10 melanoma cells	-	Suppressed cellular melanin production Inhibited tyrosinase and TRP-2 activity	[34]
Anti-hair loss	Coffee pulp Extract (0.0625 to 2 mg/mL)	Human Hair Follicle Dermal Papilla Cells (HFDPC)	-	Increased cell viability, proliferation and migration Suppressed SRD5A1, SRD5A2, and SRD5A3 gene expressions Activated Wnt/β-catenin and Sonic Hedgehog pathways	[61]
Antibacterial activity	Arabica coffee extract	Gram-positive bacteria, Gram-negative bacteria	Antimicrobial activity assays	The antimicrobial effect was independent of caffeine content MIC values for *S. aureus* and *S. epidermidis* were lower compared to Gram-negative bacteria	[62]
Antifungal activity	Spent coffee ground extract(137.50 g/mL caffeinated SCG extract, 150 g/mL of decaffeinated SCG extract)	Yeasts, dermatophytes, other Fungi	Antifungal activity assays	Antifungal activity against *C. krusei*, *C. parapsilosis*, *T. mentagrophytes*, and *T. rubrum* Reduced ergosterol content and cell wall components in *C. parapsilosis* Ultrastructural changes in fungal cells after treated	[63]
Photo- protection	Green *Coffea arabica* L. seed oil	Male hairless mice	Inducer: UV irradiation Topical Application	Reduced TEWL Reduced the number of sunburn cells and erythema	[65]

Abbreviations: α-melanocyte stimulating hormone (α-MSH); *Staphylococcus aureus* (*S. aureus*); *Staphylococcus epidermidis* (*S. epidermidis*); *Candida krusei* (*C. krusei*); *Candida parapsilosis* (*C. parapsilosis*); *Trichophyton mentagrophytes* (*T. mentagrophytes*); *Trichophyton rubrum* (*T. rubrum*).

## 9. Randomized Controlled Trials on Coffee-Based Products

Randomized controlled trials support the dermatological efficacy of coffee-derived products administered both topically and orally. Topical application of oil-in-water emulsions containing coffee lipid extract for 28 days in healthy women increased epidermal capacitance and surface lipid levels while reducing TEWL, indicating improved skin barrier integrity and hydration [66]. Likewise, oral intake of coffee polyphenol beverages extracted from green coffee beans improved skin hydration and pH balance, reduced dryness, and enhanced cutaneous blood flow during localized warming, demonstrating improved moisture retention and microcirculation [12]. Additional trials in participants with xerotic or scaly skin reported reductions in roughness and scaliness, increased smoothness, and improved skin temperature recovery after cold exposure [67]. In male subjects, four-week consumption of raw coffee bean polyphenols suppressed sodium dodecyl sulfate (SDS)-induced skin barrier damage, decreased TEWL, and improved hydration, supporting protective effects against irritant-induced dysfunction [68]. Coffee pulp extract supplementation increased skin moisture, brightness, and elasticity while reducing UV spots, wrinkles, and pore size [69]. Furthermore, topical application of *Coffea* silverskin extract gel improved hydration and relieved redness, stinging, and microcirculatory impairment following SDS irritation [70]. These findings demonstrate that both oral and topical coffee-derived formulations may enhance hydration, strengthen the skin barrier, and protect against environmental and irritant-induced stressors. Nevertheless, these randomized controlled trials are generally limited by relatively small sample sizes, short intervention durations, and heterogeneity in formulations and outcome measures, which constrain the generalizability and long-term translational implications of the reported benefits.

## 10. Safety and Regulatory Considerations

Evidence from *in vitro*, animal, and clinical studies included in this review indicates that coffee-derived materials generally exhibit a favorable safety profile within the concentrations tested, with good tolerability across biological systems. Beyond experimental findings, the long-standing global consumption of coffee beans further supports the overall safety of its phytochemical constituents [71]. However, this safety record pertains primarily to oral consumption of aqueous extracts, and systematic toxicological evaluation for topical dermatological applications remains incomplete. The reviewed randomized controlled trials (Table 6) reported no serious adverse events. Topical applications of coffee-derived materials formulations were generally well-tolerated, with only mild transient effects occasionally noted. Preclinical studies in this review demonstrating efficacy typically employed coffee extract concentrations ranging from 0.01 to 5% (*w*/*v*) for topical applications and 10–1000 μg/mL for *in vitro* studies. Establishing maximum safe concentrations and no-observed-adverse-effect levels (NOAEL) through formal dose-escalation studies is needed for regulatory submissions.

**Table 6:** Effects of coffee-derived materials on skin in randomized controlled trials.

Materials	Intervention Method	Participants	Study Duration	Control Group	Main Findings	Reference
O/W emulsions containing coffee lipid extract	Topical	Healthy female Age: 18–25 (*n* = 10)	Applied for 28 days	Blank/ Placebo	Increased epidermal capacitance and skin surface lipids Reduced TEWL	[66]
Beverage containing Coffee polyphenols (extracted from green coffee beans)	Oral	Healthy female with mildly xerotic skin Age: 25–40 (*n* = 49)	Consumed daily for 8 weeks	Placebo	Reduced skin dryness scores and TEWL Improved hydration of the SC and skin surface pH Enhanced responsiveness of skin blood flow during local warming Increased levels of free fatty acids and lactic acid in the stratum corneum	[12]
Beverage containing Coffee polyphenols (extracted from coffee beans)	Oral	Healthy female with visible scaly skin Age: 25–35 (*n* = 40)	Consumed daily for 4 weeks	Placebo	Reduction in skin scaliness and improvement in skin smoothness Improved recovery rate of skin temperature after cold stress	[67]
Beverage containing Coffee polyphenols (extracted from raw coffee beans)	Oral	Healthy male Age: 27–49 (*n* = 10)	Consumed daily for 4 weeks and irritated skin by SDS	Placebo	Suppressed the deterioration of skin barrier function caused by SDS Reduced TEWL and improve skin hydration	[68]
Drink containing coffee pulp extract	Oral	Healthy subjects Age: 35–55 (*n* = 80)	Consumed daily for 8 weeks	Placebo	Increased skin moisture, brightness and elasticity Reduced skin spots, UV spots, wrinkles and minimized pores Improved skin texture and increased collagen density	[69]
Serum containing coffee pulp extract	Topical		Applied twice daily for 4 weeks	Placebo		
Gel containing coffee silverskin extract	Topical	Healthy female with phototypes II and III Age: 18–65 (*n* = 24)	Applied twice daily for 4 weeks	Placebo	Reduced skin stinging Increased skin hydration Reduced redness, TEWL, and microcirculation impairment after SDS irritation	[70]

Abbreviations: stratum corneum (SC); *n*: sample size.

Regulatory evaluations also provide additional support. European Commission has authorized coffee leaf infusions derived from *Coffea arabica* and *Coffea canephora* as a traditional food from a third country [72,73], further supporting the long-standing history of safe use for coffee plant-derived preparations. Furthermore, the European Food Safety Authority (EFSA) has recognized that dried coffee husk (cascara) from *Coffea arabica* L. is safe under proposed conditions of use [74]. Although these approvals concern oral consumption rather than topical exposure, they provide valuable corroboration regarding the intrinsic safety of coffee plant materials as a whole.

Nevertheless, natural extracts may contain unavoidable environmental contaminants, including pesticide residues, heavy metals, mycotoxins, or solvent remnants depending on agricultural practices and extraction processes [75,76,77]. Therefore, rigorous quality control, standardized purification procedures, and compositional characterization are essential to ensure safe use in cosmetic or dermatological products. Good Agricultural Practices (GAP) and testing of final extracts using gas chromatography–mass spectrometry or liquid chromatography–tandem mass spectrometry for pesticide residues, as well as inductively coupled plasma mass spectrometry for heavy metal, are essential to ensure pesticide levels.

Topical coffee-derived materials products in dermal penetration and local bioavailability are lacking. The efficacy or toxic effects of topical coffee-derived material products depend on the skin penetration of their bioactive constituents. Coffee-derived materials exhibit distinct sensory characteristics that vary with extraction methods and processing conditions. The color of the extracts was light amber to brown, varying with concentration, and is generally compatible with cosmetic formulations. While caffeine readily penetrates the viable epidermis and dermis, polyphenols such as chlorogenic acids and mangiferin are limited by the stratum corneum barrier due to their larger size and hydrophilicity. To optimize local bioavailability, penetration enhancement strategies—including chemical enhancers and nanocarrier systems—are essential. Future research should utilize Franz diffusion cells to quantify penetration kinetics and refine formulation-driven bioavailability.

From a regulatory standpoint, cosmetic frameworks restrict the use of therapeutic or disease-related claims. However, several coffee-derived materials, including extracts obtained from different parts of the coffee plant, are listed in the International Nomenclature of Cosmetic Ingredients (INCI) as skin-conditioning agents, supporting their permitted use in cosmetic formulations [78]. Moreover, clinical studies demonstrating improvements in hydration, barrier function, and other skin parameters following oral intake of polyphenols which extract from coffee bean or coffee green bean [12,67] suggest relevance for positioning certain coffee-derived materials as skin health supplements, provided that all claims remain compliant with relevant cosmetic, nutraceutical, or food regulations. Continued research on extract standardization, contaminant control, and safety testing will be essential for advancing the responsible development of coffee-derived materials for dermatological applications.

## 11. Critical Discussion and Limitation

Despite areas of agreement across studies, several inconsistencies and unresolved questions warrant attention. Variations in botanical sources, cultivar, cultivation conditions, harvesting stage, and post-harvest processing contribute to marked differences in phytochemical composition and biological activity [79,80]. The lack of standardized extraction protocols and compositional benchmarks further complicates cross-study comparison and reproducibility. In addition, challenges in translating laboratory-effective doses from *in vitro* studies to practically achievable concentrations in humans, differences between men and women in epidemiological studies, and a notable gap between improved technical measurements and minimal visible benefits in human trials. Animal models, while useful for mechanistic exploration, are constrained by species-specific differences in skin structure, metabolism, and immune responses. Moreover, doses administered in animal studies often employ doses that would be impractical for humans to consume. Human clinical evidence remains limited and heterogeneous. Most trials typically involve small participant groups, short time periods, and measurements that may not reflect actual clinical improvement. Current evidence shows that coffee-derived materials have measurable biological activities in controlled laboratory experiments and produce improvements in some skin measurements. To advance this field, research efforts should prioritize rigorous human clinical studies, understanding how these compounds behave in the body, and direct comparisons with proven treatments. While controlled laboratory studies provide convincing evidence of biological activity, the translational gap between experimental findings and robust clinical efficacy remains substantial.

## 12. Conclusions

This review synthesizes current evidence on the dermatological applications of coffee-derived materials from diverse sources including coffee beans and by-products such as leaves, silverskin and spent grounds. We assessed evidence quality across *in vitro* studies, animal models, and human clinical trials, examined dose–response feasibility and bioavailability constraints, evaluated methodological limitations particularly in observational epidemiological studies, and identified critical translational barriers and research gaps. Coffee extracts and their derivatives exhibit a broad range of dermatological activities mediated through antioxidant, anti-inflammatory, and regenerative mechanisms. Studies have documented their capacity to modulate key molecular pathways, including ROS scavenging [38,81], MAPK/AP-1 signaling [17], and NLRP3 inflammasome [45] modulation in experimental models of skin disorders. Evidence from *in vitro*, animal models, and preliminary human trials indicates potential benefits in improving skin hydration, supporting barrier function, facilitating tissue repair, and reducing damage from oxidative and environmental stressors.

While preclinical investigations provide encouraging evidence for antioxidant, anti-inflammatory, and photoprotective properties, the translational trajectory from bench to bedside remains obstructed by significant methodological heterogeneities, a pervasive lack of phytochemical standardization, and high-powered, longitudinal clinical trials. Current research presents some methodological variations across studies, differences in phytochemical standardization approaches, and opportunities for more extensive clinical validation through larger, longer-duration trials. In addition, studies largely focus on isolated bioactive compounds or single-extract applications, whereas the interactions among coffee-derived metabolites, formulation stability, and dose–response relationships require further clarification. Additional investigation into the pharmacokinetic profiles, skin penetration characteristics, and precise molecular mechanisms of specific coffee phytochemicals—as well as their potential synergistic or additive interactions within cosmetic formulations—would advance understandings of their dermatological applications. To support the continued development of coffee-derived bioactives for cosmeceutical and potential pharmaceutical applications, several research directions appear promising. These include bioassay-guided fractionation approaches to identify and characterize the most active constituents, development of enhanced delivery systems to optimize bioavailability, and establishment of standardized analytical methods for extract characterization and quality control. Considerations for clinical translation include establishing quality-control standards for extract composition, ensuring batch-to-batch consistency, and evaluating allergenicity or irritancy in topical use will be important.

Bridging these evidence gaps through standardized characterization and comparative effectiveness research against some well-known ingredients (such as vitamin C) for skin disorders would provide valuable context for their clinical utility. Such comparative effectiveness research, combined with standardized characterization methods and appropriately powered clinical trials, would help clarify the role of coffee-derived materials as functional ingredients in clinical dermatology and wound care applications. Coffee-derived materials have been explored for incorporation into wound-healing scaffolds and bioactive dressings; however, further investigation is required in this area to fully understand their interactions within biological systems.

Coffee-derived materials and compounds represent scientifically interesting bioactive ingredients with demonstrated biological activities in experimental systems and preliminary evidence of dermatological benefits. The field would benefit from continued methodological refinement, enhanced standardization, and rigorous clinical validation to fully realize their potential as evidence-based dermatological ingredients.

## Data Availability

No new data were created or analyzed in this study.
